# HIV and the gut: implications for HIV persistence, immune dysfunction and cure strategies

**DOI:** 10.3389/fimmu.2025.1650852

**Published:** 2025-09-18

**Authors:** Jillian S. Y. Lau, Sharon R. Lewin, Sushama Telwatte

**Affiliations:** ^1^ Department of Infectious Diseases, The University of Melbourne at the Peter Doherty Institute for Infection and Immunity, Melbourne, VIC, Australia; ^2^ Victorian Infectious Diseases Service, Royal Melbourne Hospital at the Peter Doherty Institute for Infection and Immunity, Melbourne, VIC, Australia; ^3^ Monash Infectious Diseases, Monash Health, Clayton, VIC, Australia; ^4^ Department of Infectious Diseases, Alfred Hospital and Monash University, Melbourne, VIC, Australia

**Keywords:** HIV reservoir, gut-associated lymphoid tissue (GALT), HIV persistence, mucosal immunity, latency reversal, gene and cell therapies, HIV cure strategies, antiretroviral penetration

## Abstract

The intestinal immune compartment plays a central role in HIV pathogenesis, serving as an early site for viral replication and a significant reservoir for latent infection. Despite the success of antiretroviral therapy (ART) in suppressing plasma viremia, HIV persists indefinitely in latently infected cells, commonly found in the intestinal tract due to its unique immunological and structural environment. Targeting HIV-infected cells that persist in the intestinal tract is an important consideration for therapeutic strategies and is also important when considering an HIV cure. This review describes the therapeutic approaches aimed at addressing HIV persistence in the intestinal tract, or gut. We provide a brief overview of mechanisms underlying reservoir formation and maintenance, discuss the challenges posed by gut-specific factors, and examine emerging strategies, including latency reversal agents, immune modulation, gut-targeted ART, and novel delivery systems. This review will focus on contemporary advances in knowledge in this space, gaps in the literature and areas for future research focus.

## 1 Introduction

While antiretroviral therapy (ART) effectively suppresses plasma viral levels, it fails to eliminate latent reservoirs of HIV, particularly in gut associated lymphoid tissue (GALT) (1). The gut serves as a major anatomical reservoir due to the high density of activated, HIV-susceptible CD4^+^ T cells expressing the major co-receptors for HIV, CCR5 and CXCR4 (2–4), unique lymphocyte trafficking patterns, variable antiretroviral (ARV) penetration (5–8), and an immunoregulatory environment. The gut contains over 85% of lymphoid tissue and more than 90% of all lymphocytes (9), making it a critical compartment in HIV pathogenesis and persistence.

The intestinal immune system comprises inductive sites (e.g., mesenteric lymph nodes and Peyer’s patches), where adaptive immune cells (CD4+ T cells, CD8+ T cells and B cells) are initially activated and differentiate; and effector sites (e.g., lamina propria and epithelium), where differentiated immune cells mount mucosal defence (10, 11).

A critical component within this compartment, GALT, plays a key role in antigen sampling and comprises multi-follicular structures like Peyer’s patches in the small intestine, isolated lymphoid follicles (ILFs) that are dispersed throughout the small and large intestines (10, 11), as well as sites such as the appendix (12) and rectal lymphoid tissue (13, 14). The abundance of activated CD4+ T cells, with a predominantly memory (CD45RO+) phenotype that also express the HIV co-receptors CCR5/CXCR4 (2–4) coupled with the extensive mucosal surface area at this site, renders GALT especially susceptible to HIV infection (4). Early studies revealed a profound depletion of CCR5^+^ memory CD4+ T cells following acquisition of HIV (15), particularly in effector sites of the lamina propria (16, 17), where CCR5-expressing memory CD4+ T cells were rapidly lost (18). As shown in both SIV and HIV infection, there is a preferential depletion of CD4+ T cell in mucosal-associated lymphoid tissue compared with peripheral blood that is more severe in mucosal tissues than in peripheral blood, and disproportionately affects Th17, Th22 and other immune-regulatory subsets essential for maintaining mucosal barrier function (18–25). Notably, other contemporaneous studies suggested that the virus spares long-lived naïve and central memory CD4+ T cells, which may replenish depleted effector cells (26, 27). Countering the prevailing hypothesis of direct viral cytopathicity (28), these studies suggested that chronic immune activation may be the primary driver of progressive CD4+ T cell loss (26, 27). Nonetheless, HIV persistence in the intestinal immune compartment poses a formidable barrier to achieving remission or eradication of HIV (10–14). In this review, we examine the mechanisms underpinning HIV persistence in the gut and explore emerging therapeutic strategies tailored to this complex immune compartment.

## 2 Mechanisms of HIV reservoir persistence in the gut

### 2.1 Structural and cellular factors

**Figure 1:**
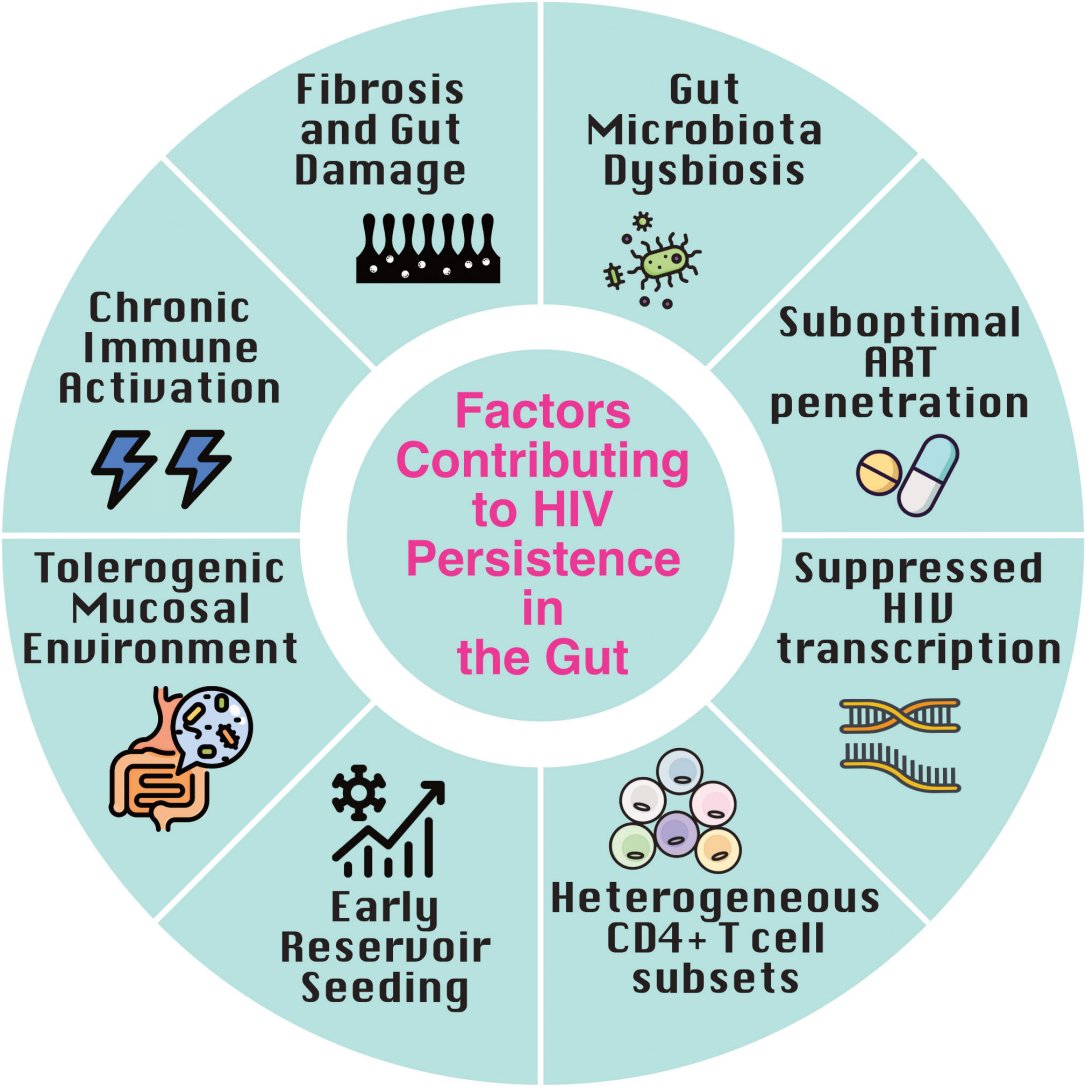
Factors contributing to HIV persistence in the gut. Multiple factors converge in the gut to promote HIV latency and persistence despite antiretroviral therapy (ART). Early reservoir seeding during acute infection, combined with a tolerogenic mucosal environment and immune-evasive tissue-resident memory (TRM) cells, establishes a durable reservoir. Chronic immune activation driven by microbial translocation, along with gut-associated lymphoid tissue (GALT) fibrosis, impairs immune clearance. Heterogeneous CD4^+^ T cell subsets, suppressed transcriptional activity, suboptimal ART penetration, and gut microbiota dysbiosis further reinforce viral persistence and immune evasion within this immunologically distinct tissue.

HIV disrupts the gut’s three key barriers: the microbial barrier (commensal bacteria) (29), the mechanical barrier (tight junctions between epithelial and endothelial cells) (30, 31), and the immunologic barrier (mucosal lymphocytes, mesenteric lymph nodes, and cytokines) (17, 32, 33). These disruptions drive chronic immune activation and facilitate viral persistence (**Figure 1**).

Anatomical sites vary in immune cell composition (34), tolerogenic features (35), and the transcriptional landscape of the HIV reservoir (36), resulting in differential viral burden (37, 38) and tissue-specific responses to latency-targeting interventions (39). Microbial sensing by gut macrophages leads to IL-1 production and downstream secretion of regulatory cytokines (retinoic acid, IL-10, TGF-β), which foster a tolerogenic environment (40, 41). The gut, in particular, is a key site of immune tolerance and a primary reservoir for HIV, where constant exposure to microbiota-derived signals drives the differentiation and maintenance of HIV-susceptible CD4^+^ T cell subsets, including regulatory T cells (Tregs), Th1, Th2, Th17, and Th22 cells (42–45). Many of these cells express high levels of HIV co-receptor CCR5 (43), rendering them highly susceptible to infection during acute and chronic phases. Th17 and Th22 subsets, which are enriched in the lamina propria and play critical roles in maintaining mucosal barrier integrity and microbial defense, are particularly vulnerable targets (25, 46, 47). Recent findings suggest that CCR6-expressing CD4^+^ T cell subsets, including Th17, Th1Th17, and CCR6^+^CCR4^-^CXCR3^-^ cells, represent a substantial and preferentially infected reservoir in the gut, enriched for replication-competent HIV due to their mucosal localization, memory phenotype, and high susceptibility to infection (47–49).

Another pivotal cellular reservoir in the gut are tissue-resident memory T cells (TRM), which differ from circulating memory T cells in both phenotype and function. TRM are long-lived, non-recirculating cells that localize to mucosal barrier sites and are poised for rapid immune responses upon local antigen re-encounter (50). In the gut, regionalized signaling within the intestinal microenvironment supports the maintenance of two phenotypically distinct TRM states: terminally differentiated TRM cells localized to the upper villus, and progenitor-like TRM residing in the lower villus (51). CD8^+^ TRMs acquire CD103 expression under the influence of local TGF-β and IL-10 (52, 53), while most CD4^+^ TRMs lack CD103 (54). Despite this, both gut-resident and circulating CD103^+^ CD4^+^ T cells share a gene expression profile enriched for HIV DNA but exhibit low levels of RNA transcription per provirus, consistent with latent infection (55). The shared molecular mechanisms, including reduced expression of ribosomal proteins and components of mRNA processing and transcriptional machinery, suggest common mechanisms of proviral silencing (55). Functionally, TRMs express elevated levels of inflammatory and cytotoxic genes (54, 56, 57), enabling rapid effector responses. However, they also upregulate inhibitory markers such as PD-1 and CD101, which constrain proliferation and IL-2 production (54, 58). These dual attributes, activation readiness and functional suppression, highlight their role as both sentinels of mucosal immunity and long-lived HIV reservoirs that may be less accessible to immune clearance or ART penetration (54, 55). These features may contribute to immune evasion and present barriers to latency reversal strategies, underscoring the need for targeted approaches that can overcome the unique functional and transcriptional constraints of the gut-resident reservoir.

### 2.2 Persistent immune activation and inflammation fuel HIV persistence

Chronic immune activation is a hallmark of HIV infection and a key driver of reservoir persistence (**Figure 1**). Persistent infection is characterized by a dynamic equilibrium in which ongoing immune activation coexists with regulatory mechanisms that limit immunopathology but may also permit viral persistence (43). Elevated levels of pro-inflammatory markers such as IFN-γ, IL-6, IP-10, and indoleamine 2,3-dioxygenase promote CD4^+^ T cell susceptibility and sustain inflammatory cycles (59–62). A higher proportion of activated and proliferating T cells are found in the gut compared to peripheral blood (22, 63), contributing to reservoir maintenance. This activation is largely triggered by HIV-mediated damage to the intestinal epithelium, leading to microbial translocation- the leakage of bacterial products like lipopolysaccharide (LPS) into circulation- which fuels systemic immune activation in both people with HIV (PWH) and SIV-infected macaques, and strongly predicts disease progression (29, 64–66). Fungal translocation, particularly of (1→3)-β-D-glucan, further amplifies inflammation via pattern recognition receptor signaling, and remains elevated despite ART, contributing to gut damage and disease progression (67, 68).

Although ART effectively suppresses plasma viremia, it does not fully restore gut epithelial integrity or microbiome diversity (22, 67, 69, 70). Consequently, immune activation and microbial translocation markers persist, undermining immune homeostasis and facilitating continued HIV persistence (67, 71–73). Ongoing inflammation also recruits new target cells for infection, reinforcing the reservoir despite viral suppression (74). Together, these features create a uniquely permissive environment in the gut for HIV latency and immune evasion, even under sustained ART.

### 2.3 Gut microbiota, dysbiosis and microbial translocation

Recent findings suggest that gut microbiota composition may modulate reservoir size and immune control. A recent germ-free humanised mouse model demonstrated the role the microbiome plays in HIV persistence, with lower levels of HIV replication in plasma and tissues of germ-free mice, depleted of their resident microbiota, compared with conventional humanised mice (75). In a study of HIVconsv immunogen (conserved regions of HIV-1 Gag, Pol, Vif, and Env) and the histone deacetylase inhibitor (HDACi) romidepsin, a latency reversing agent (LRA); individuals with higher baseline gut *Bacteroidales: Clostridiales* ratios showed smaller HIV reservoirs and more sustained control of viremia (76). *Bacteroidales* species, known producers of immunomodulatory metabolites like short-chain fatty acids, may influence T cell function and mucosal immunity (77), implicating microbial dysbiosis as both a consequence and modulator of HIV persistence. Finally, a pilot randomised controlled trial of repeated faecal microbiota transplantation in PWH found no differences in biomarkers of inflammation and bacterial translocation between treatment and control groups, but no comparisons of HIV reservoirs were conducted in this study (78).

The presence and mechanism of a causal link between gut barrier dysfunction, microbial translocation, systemic inflammation and HIV persistence is not well understood (79, 80) and warrants further study.

### 2.4 ARV penetration in the gut: barriers to reservoir elimination

Reduced antiretroviral drug penetration in the gut may pose a challenge to the elimination of the HIV reservoir in this compartment. Studies have shown that mucosal tissue penetrance varies with antiviral agents; due to a range of intrinsic and extrinsic factors (protein binding, molecular size, lipophilicity, ionization, and blood perfusion), physical barriers, as well as efflux and uptake transporter expression (5); leading to levels of some ARVs, such as the integrase strand transfer inhibitor (INSTI) dolutegravir and non-nucleoside reverse transcriptase inhibitor rilpivirine, falling below therapeutic thresholds in gut tissues compared to peripheral blood (5, 81). Consistent with these findings, individuals with lower tissue drug concentrations exhibit higher HIV transcription at these sites despite systemic viral suppression (82, 83). Some studies have even suggested that the HIV reservoir is constantly replenished by low-level virus replication in lymphoid tissue despite undetectable viral RNA in plasma (81, 84), although this remains controversial and has been strongly challenged by others (85, 86). Nonetheless, recent pharmacokinetic studies of newer, long-acting (LA) injectable drug formulations also demonstrate lower gut tissue penetrance (6), despite their long half-life and superiority over combination tenofovir disoproxil fumarate/emtricitabine in the setting of HIV pre-exposure prophylaxis (87, 88). In a Phase I study of the INSTI cabotegravir-LA, rectal concentrations of the drug were <8% of the corresponding plasma concentration (6). In contrast, drug concentrations of LA injectable rilpivirine in rectal tissue were found to exceed plasma levels *in vivo* and showed a dose-dependent antiviral effect *ex vivo (* 7), suggestive of more durable mucosal protection. A recent study of LA cabotegravir plus rilpivirine found that some individuals continued to shed HIV-1 RNA in rectal secretions despite plasma suppression, and rectal rilpivirine levels, though above the protein-adjusted EC_90_, did not correlate with viral shedding, suggesting that drug exposure alone may not fully suppress HIV transcription or replication in the gut (8). Other factors including ongoing immune activation, suboptimal immune control and high burden of latently infected cells in the gut (89) likely also contribute to observed persistent transcription and compartmentalized viral replication. Notably, while genetically intact HIV DNA can be detected in tissues including the ileum, colon, and rectosigmoid, the presence of markers of transcription completion and protein production (polyadenylated and multiply-spliced HIV transcripts) are infrequently detected in gut and female genital tract tissues in virally-suppressed PWH (36, 38). These findings underscore the complexity of achieving complete viral suppression in different mucosal compartments and the need to consider both drug distribution and local tissue factors when evaluating the efficacy of long-acting ART formulations.

## 3 Emerging therapeutic approaches

### 3.1 Latency modulating agents

**Figure 2:**
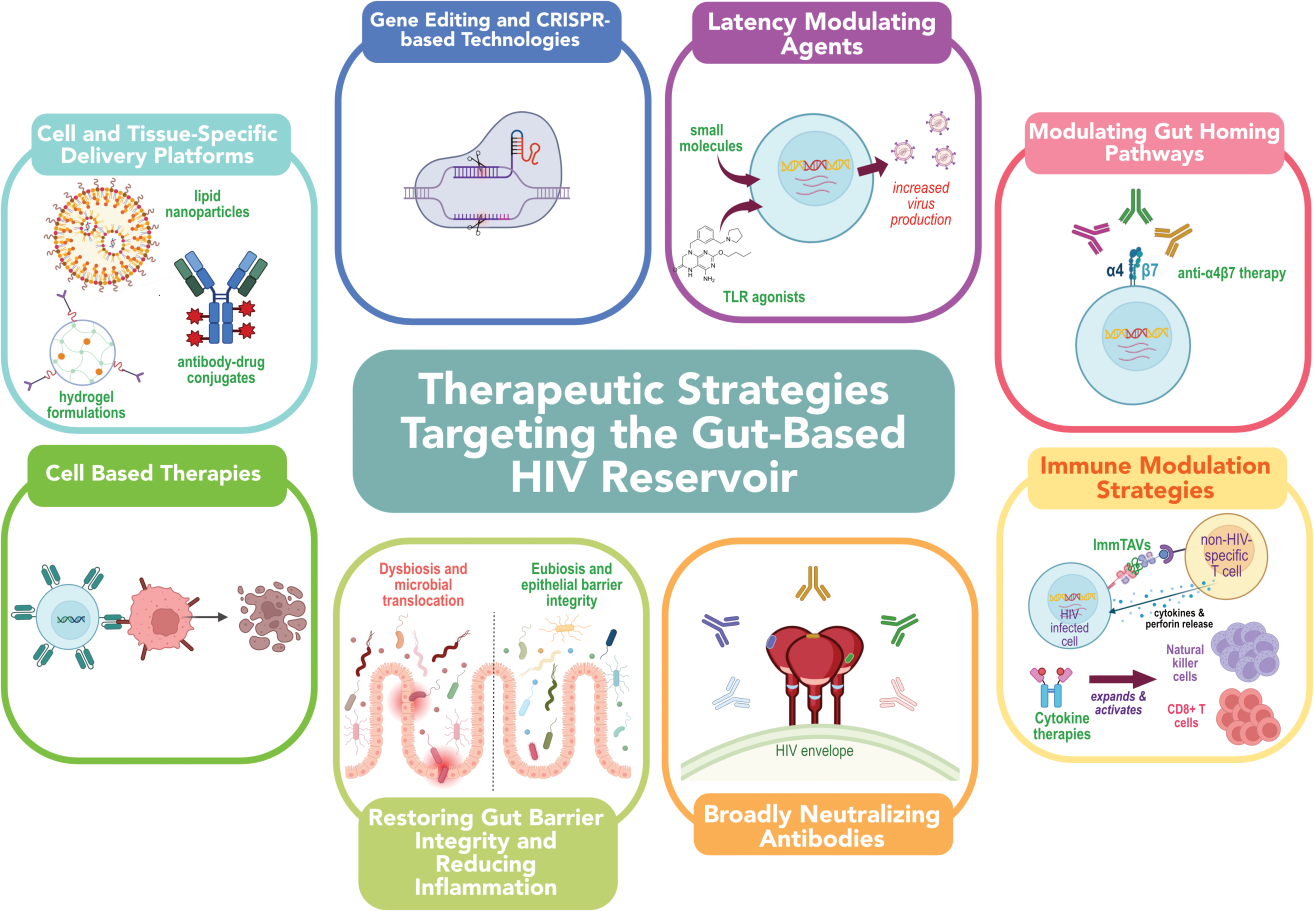
Emerging therapeutic strategies for targeting the HIV reservoir in the gut. Strategies include: (1) latency-modulating agents to induce viral gene expression; (2) gut-homing pathway modulation via integrin-targeting antibodies; (3) immune-based therapies to enhance clearance of infected cells; (4) broadly neutralizing antibodies to block infection and mediate cytotoxicity; (5) interventions to restore gut barrier integrity and reduce inflammation; (6) cell-based therapies, (7) gene-editing and CRISPR-based approaches; and (8) targeted delivery platforms such as nanoparticles and antibody-drug conjugates to improve localization and efficacy of therapeutics within the gastrointestinal tract. Together, these approaches aim to overcome anatomical and immunological barriers to HIV cure.

Latency reversal agents (LRAs) aim to reactivate latent HIV to increase virus transcription, protein expression and virion production (**Figure 2**), thereby making infected cells visible to the immune system for immune-mediated clearance (90). A diverse array of transcription activating LRAs have been assessed both *in vitro, ex vivo* and *in vivo* for their ability to reactivate HIV transcription with varying levels of success- a topic that has been comprehensively reviewed elsewhere (90–93).

*Ex vivo* analysis of LRAs demonstrate that agents that can induce HIV transcription in peripheral blood may not exert the same magnitude of effect in gut tissues (39). Notably, even when HIV transcription is induced, the translation of viral proteins or production of virions- crucial for immune recognition- may be limited, raising questions about the functional efficacy of many LRAs, particularly in tissue-resident cells. As such, the distinct immune milieu of the gut (**Figure 1**) may necessitate the development of gut-specific LRAs or combination strategies that improve both HIV reactivation and immune-mediated clearance in this compartment.

Emerging immunomodulatory LRAs, such as Toll-like receptor (TLR) agonists, TLR-1/2, TLR7, and TLR9 (94–97), have demonstrated potential in both reactivating latent virus and modulating immune responses in humans and non-human primates. TLR agonist stimulation induces an activated plasmacytoid dendritic cell phenotype, with increased expression of TNF-α, IFN-α, interferon regulatory genes and restriction factors that contribute to an increased HIV-specific T cell response (98). Treatment with the TLR9 agonist lefitolimod results in induction of an interferon stimulated gene signature consistent with potent IFN-α induction but without concomitant excessive inflammation in the gut mucosa (95, 99), suggesting this treatment could exert beneficial effects in the gut. The combination of TLR7 agonist vesatolimod (GS-9620) and broadly neutralizing antibody PGT121 led to lower levels of HIV DNA in lymph nodes of treated SHIV-SF162P3-infected rhesus monkeys at week 120 (100) and decreases in intact proviral DNA in peripheral CD4+ T cells during ART coupled with a delay in viral rebound during ART interruption in a phase 1b clinical trial (101). More recently, dual TLR7/8 agonists have demonstrated enhanced latency reversal and immune activation compared to single agonists, resulting in greater reductions in the inducible HIV reservoir and improved control of viral rebound in preclinical (*ex vivo* PBMC/cell line) models (102).

While considerable progress has been made in the development of latency reversal strategies, there is limited evidence supporting the efficacy of LRAs in the gut. Latency reversing effects in colonic and rectal tissue have been demonstrated in clinical trials of the HDACis panobinostat and vorinostat (103, 104), and the TLR9-agonist lefitolimod (99), however in the case of vorinostat, this effect less than that noted in peripheral blood cells. Notably, few clinical trials investigating LRAs have routinely conducted biopsies of gastrointestinal tissue.

An alternative latency-modulating approach under investigation, the “block and lock” strategy, seeks to drive HIV into a long-lived, transcriptionally silent state. This has been studied with agents such as bromodomain-containing protein 4 (BRD4) modulators (105), Heat shock protein 90 (HSP90) inhibitors (106–108), LEDGINs (109, 110), Jak-STAT inhibitors (111, 112), HIV-1 Tat inhibitor didehydro-cortistatin A (113). Notably, didehydro-cortistatin A has a favorable pharmacokinetic profile, stability, activity in the absence of ART, and the ability to cross the blood–brain barrier, making it a particularly attractive candidate (113, 114). Given the gut’s role as a major site of persistent HIV transcription, strategies that can durably suppress proviral expression in tissue-resident immune cells could be critical for achieving long-term remission. The distinct immunologic landscape of the gut underscores a critical gap in current HIV cure strategies, highlighting the need for more trials to evaluate the impact of interventions on gut reservoirs and to develop gut-targeted or combination approaches that both induce HIV transcription and enhance immune-mediated clearance.

### 3.2 Modulating gut-homing pathways through antibody-based interventions

CD4+ T cells migrate into gastrointestinal tissues by engaging α4β7 integrin, expressed on their surface, with MAdCAM-1, a key adhesion molecule found on the gut endothelium (115, 116). HIV gp120 binds to this gut-homing integrin (117), enhancing the susceptibility of α4β7+CD4+ T cells to HIV infection (118). Therefore, targeting α4β7, which facilitates the trafficking of HIV-infected cells to the gut (118–121), represents a novel strategy to disrupt reservoir formation and persistence.

Treatment with anti-α4β7 therapy [vedolizumab (VDZ)] in PWH on ART with concomitant inflammatory bowel disease (IBD) has demonstrated promise in attenuating the formation of lymphoid aggregates within the gut (122), which are known to serve as key sanctuary sites for maintaining viral reservoirs (123–126). While VDZ resulted in sustained virologic control in one study of macaques infected with an attenuated strain of SIV, SIVmac239 (127), this finding triggered some controversy as the SIV strain used in the study had a stop codon in the Nef coding region (128). Subsequent studies in macaques infected with SIVmac251 (129) and in PWH on ART (130), could not replicate this observation. Nonetheless, a recent clinical trial suggested that the level of α4β7 blockade may inversely correlate with HIV DNA levels (131), highlighting a potential role for integrin-targeting strategies in reducing viral reservoirs, though further research is needed to explore their utility in combination cure approaches.

In addition to α4β7, other trafficking molecules such as CCR9, which is important for gut homing particularly in Th17 cells during HIV infection (132) and MadCAM-1 are being explored as potential therapeutic targets in reducing inflammation (133) that could provide gut-selective options that avoid systemic immunosuppression.

### 3.3 Immune modulation strategies to enhance HIV reservoir clearance

Immune modulation strategies aim to restore antiviral immunity, reduce chronic inflammation, and enhance the immune system’s capacity to recognize and eliminate infected cells within mucosal tissues (134). Checkpoint blockade targeting inhibitory receptors (e.g., PD-1, CTLA-4, LAG-3, TIGIT) has shown promise in reversing T cell exhaustion and enhancing HIV-specific CD8^+^ T cell responses in preclinical studies [reviewed elsewhere (134)]. These approaches may synergise with LRAs (135), but systemic administration risks immune-related adverse events (136), highlighting the need for gut-targeted strategies.

Cytokine-based therapies, such as IL-15 superagonists may enhance mucosal effector responses (137, 138). The IL-15 superagonist N-803 (**Figure 2**) modestly reduced inducible HIV in peripheral blood mononuclear cells (PBMC) in a Phase I trial, alongside natural killer (NK) cell expansion (137). In SIV-infected macaques, N-803 increased CD8^+^ T cell and NK cell activation and trafficking to lymphoid and mucosal tissues (139, 140), highlighting its potential to bolster immune clearance mechanisms in mucosal reservoirs. However, the effects of N-803 in the human gut remain largely uncharacterized, underscoring the need for dedicated studies to evaluate its impact on the gut HIV reservoir.

Emerging bispecific platforms like ImmTAVs- soluble, engineered T-cell receptors (TCRs) fused to anti-CD3, redirect polyclonal CD8^+^ T cells to eliminate HIV-infected CD4^+^ T cells presenting low levels of HIV antigen (141). Given the low-level HIV Gag expression detectable in gut tissues of ART-suppressed individuals (142, 143), ImmTAVs may offer a potent strategy for mucosal reservoir clearance. Although current constructs are HLA-restricted and untested in gut tissue, their efficacy in solid tumours (144) supports their translational potential. Advantages of this strategy include: (i) targeting cells expressing very low antigen levels; (ii) bypassing exhausted HIV-specific T cells; and (iii) compatibility with combination therapies.

Immune-based approaches must carefully balance antiviral activity with the preservation of mucosal barrier function and limitation of inflammation-induced damage. Refinement of these strategies is ongoing, aiming to enhance antiviral responses while preserving mucosal integrity. For instance, targeted delivery mechanisms, such as nanoparticle formulations and antibody-drug conjugates (Section 2.7) are being explored to localize immune modulation to mucosal tissues and reduce systemic toxicity. Combinatorial approaches, such as pairing cytokines like IL-15 or IL-21 with checkpoint blockade (145) or probiotic therapy (146), are being optimized to enhance effector cell function without inducing excessive inflammation. Additionally, advances in cancer immunotherapy, such as checkpoint blockade targeting myeloid-derived suppressor cells (MDSCs) to overcome their immunosuppressive effects and enhance the efficacy of immune checkpoint inhibitors (ICIs) and adoptive cell therapies (147), may inform combinatorial strategies for targeting the HIV reservoir. These emerging approaches reflect a broader shift toward precision immunotherapies that are tailored to the distinct immunologic and structural characteristics of mucosal tissues- an important step toward more effective strategies for targeting and eliminating HIV reservoirs in these challenging anatomical sites.

### 3.4 Broadly neutralizing antibodies

Broadly neutralizing antibodies (bNAbs) can induce direct viral neutralization and immune responses through antibody-dependent cellular cytotoxicity (ADCC) (148–151). Despite some promising data showing that the combination of bNAbs 3BNC-117 and 10–1074 can significantly delay viral rebound following ART interruption (152), concomitant decreases in the size of the viral reservoir have not been demonstrated. A recent proof-of-concept study using a 10–1074 formulated for topical vaginal application demonstrated that mucosal delivery of potent bNAbs provided protection against repeated cell-associated SHIV162P3 vaginal challenge in non-human primates (153). Clinical trials are currently ongoing to assess the effect of long-acting bNAbs on the tissue-resident viral reservoirs (154).

### 3.5 Restoring gut barrier integrity and reducing inflammation

A wide array of therapeutic strategies has been explored to target the gut microbiome in PWH, aiming to reduce persistent inflammation and immune dysfunction despite effective ART (**Figure 2**).

Antibiotics, administered experimentally, have shown mixed outcomes, with some studies in nonhuman primates (NHPs) suggesting reduced gut inflammation and altered susceptibility to SIV infection, however concerns remain about long-term dysbiosis and resistance (155) that may compromise gut barrier integrity. In human trials examining antibiotics as a possible modality to ameliorate persistent immune dysfunction in ART-suppressed PWH, neither rifaximin nor cotrimoxazole treatment altered bacterial translocation (156, 157). Although antibiotics can influence the gut microbiome in PWH (155), their use warrants caution due to broad microbial disruptions and the risk of antimicrobial resistance.

Prebiotics and probiotics have demonstrated modest benefits on immune markers in some exploratory trials (158–160), but larger controlled studies in children infected with HIV, and ART naive adults with HIV failed to show consistent improvements in gut dysbiosis, immune recovery or reduction in inflammatory biomarkers (161–163). Despite some reported benefits, including potential improved gut barrier integrity (164), current prebiotic and probiotic formulations lack sufficient evidence and regulatory oversight to support their use in PWH (155), highlighting the need for more targeted, next-generation approaches.

More recently, attention has shifted to postbiotics and live biotherapeutic products (LBPs) driven by progress in the treatment of *Clostridium difficile (* 165, 166). Approaches include delivery of targeted bacterial consortia, designed to restore or improve gut microbiota composition, and microbial metabolites like butyrate (167), that could have anti-inflammatory or immune-modulating effects (159, 168) and enhance epithelial barrier function (169), however their immunological efficacy in the setting of HIV remains under investigation. Faecal microbiota transplantation (FMT) is under investigation as a strategy to reverse HIV-associated gut dysbiosis, with early-phase trials demonstrating transient donor engraftment (170), enhanced microbial diversity (171), and indications of reduced gut epithelial damage (172). While these findings highlight FMT’s potential to modulate the gut microbiota in PWH, evidence for its impact on systemic inflammation and HIV persistence remains inconclusive, underscoring the need for further research into microbiota-targeted interventions to address HIV-driven immune dysfunction.

Therapies aimed at reducing systemic inflammation or restoring gut barrier integrity in PWH target key drivers such as microbial translocation, residual viral replication, and immune dysregulation. Investigational approaches include anti-inflammatory agents (e.g., statins (173–176)); immunomodulators like IL-1β blockers (177, 178), IL-6 blockers (177, 178), tumour necrosis factor a (TNFa) blockers (179, 180), toll-like receptor 4 (TLR4) antagonists (181), PPAR agonists (182, 183), or Janus kinase (JAK) inhibitors (179, 180); farnesoid X nuclear receptor (FXR) agonists (184) sulfonamide drugs (185); and gut-tropic agents such as GLP-2 analogue teduglutide (Clinical Trial: NCT02431325). Apolipoprotein A-I (apoA-I) mimetic peptides bind bioactive lipids and endotoxin (LPS) to exert an anti-inflammatory effect (186). Previously investigated as a treatment modality for cardiovascular disease and cancer (187, 188), recent work in humanized mouse models of HIV infection suggest that these peptides can reduce levels of proinflammatory proteins, such as ADAM17, that contribute to both systemic and gut inflammation (189).

While several of these strategies have demonstrated reductions in biomarkers of inflammation, their capacity to meaningfully improve immune function or reduce clinical comorbidities in PWH has yet to be definitively established.

Together, these findings underscore the complexity of therapeutically targeting the gut in PWH and highlight a critical need for rigorously designed, mechanistically informed studies to identify microbiota-directed or gut-specific interventions that can durably reduce inflammation, restore mucosal integrity, and ultimately contribute to HIV remission or cure strategies.

### 3.6 Therapeutic vaccines to restore gut immunity and reduce mucosal inflammation

Therapeutic vaccines represent a promising avenue for enhancing gut immunity in PWH. By targeting the gut mucosa, these strategies aim to restore immune function, reduce inflammation, and improve overall health outcomes in PWH. Several approaches are under investigation, including intranasal or mucosal vaccines adjuvanted with IL-13Rα2 blockers, which have been shown to enhance mucosal CD8^+^ T cell responses in gut-associated lymphoid tissues (190). Other strategies involve dendritic cell-targeted vaccines designed to induce durable HIV-specific immunity at mucosal sites (191, 192), oral vaccines using recombinant *Lactococcus lactis* expressing HIV antigens which have demonstrated the ability to elicit mucosal immune responses (193), and mRNA-based vaccines that promote polyfunctional T cell responses within the gastrointestinal tract.

While prophylactic mRNA HIV vaccines are progressing (e.g., NCT05001371, NCT05414786, NCT05217641), therapeutic HIV vaccine development remains limited (194). Unlike prophylactic strategies that focus on eliciting envelope-specific neutralizing antibodies (195), therapeutic vaccines must induce strong, Gag-specific polyfunctional CD8+ T cell responses (196–198). To date, only a limited number of therapeutic vaccine candidates have progressed beyond preclinical evaluation in mouse and non-human primate models (199–201). Therapeutic vaccines, such as ALVAC-HIV/Lipo-6T/IL-2 (202), Vacc-4x (203), and HIVACAT T-cell immunogen-based vaccines (204) have shown promise in enhancing viral control in the absence of ART, and may even help overcome the impact of gut microbiota depletion on IFNγ-producing T-cell responses (205). However, their standalone efficacy has been limited, therefore combination strategies may be necessary to achieve sustained viral remission and counteract immune dysfunction originating in the gut. A major gap remains in understanding how to direct antigen-specific immune responses to the gut and how to measure functional improvements in gut immune health after therapeutic vaccination. While clinical translation is ongoing, these approaches represent promising adjuncts to antiretroviral therapy by addressing the immunologic damage and inflammation that persist in the gastrointestinal mucosa.

### 3.7 Gene and cell-based therapeutic strategies

Gene editing technologies, particularly CRISPR-based approaches, have emerged as promising tools for targeting persistent HIV reservoirs. These strategies are especially relevant to GALT, where therapeutic interventions must be able to access, persist, and function effectively.

Viral-directed approaches aim to excise integrated provirus (206), durably silence transcription (207) or activate latent proviruses to enhance clearance (208). Recent efforts emphasize the need for delivery systems that achieve effective biodistribution within lymphoid tissues, where the majority of the reservoir resides (209, 210). The CRISPR-based therapy EBT-001, delivered by adeno-associated virus (AAV), achieved broad biodistribution in lymphoid tissues and demonstrated evidence of proviral cutting in preclinical simian immunodeficiency virus (SIV) models (206). Its HIV counterpart, EBT-101, was recently shown to be safe in a first-in-human clinical trial, although viral rebound occurred following analytical treatment interruption, highlighting the need for further refinement (211).

Host-directed approaches aim to render target cells resistant to infection or to enhance antiviral immunity in mucosal compartments (212), CCR5 remains a leading gene-editing target, with multiple studies demonstrating disruption of CCR5 using zinc finger nucleases (ZFNs), TALENs, and CRISPR is feasible *in vitro, ex vivo*, and *in vivo* (212–215). These approaches provide proof-of-concept for durable resistance to HIV infection and, importantly, could protect gut-homing memory CD4+ T cells from reinfection. and engineering of host cells resistant to infection (212).

Complementary to gene editing, cell-based therapies are advancing in parallel. In nonhuman primate models, stem-cell-derived CAR T cells demonstrated superior persistence, tissue trafficking, and antiviral activity, reinforcing their potential in mucosal immune compartments (216). Notably, a macaque study demonstrated that hematopoietic stem cell (HSC)-derived CAR T cells engraft and persist within tissue-associated HIV reservoirs, including GALT, where they maintained proliferative capacity and antiviral activity (216). Similarly, CAR/CXCR5 T cells showed modest presence in gut tissues (ileum, rectum) alongside sustained reductions in viral RNA within lymphoid follicles, underscoring both the potential and current limitations of tissue penetration (217). Early-phase clinical trials, including CAR T-cell therapies targeting gp120 (218), are underway, though mapping gut homing and durability of responses remain critical next steps.

Together, these gene- and cell-editing strategies underscore the potential to overcome the unique barriers posed by gut reservoirs, where persistence, immune evasion, and tissue accessibility converge, positioning the gut as a critical testing ground for next-generation HIV cure interventions.

### 3.8 Cell and tissue-specific delivery platforms

Targeting therapeutic agents directly to gut-associated lymphoid tissue represents a major challenge and opportunity in HIV cure research. The anatomical and immunological complexity of the gut (9, 10), coupled with its unique barriers to drug penetration and immune accessibility (89), necessitates the development of innovative delivery systems designed to enhance the localization, uptake, and activity of targeted therapeutics (219, 220). Nanoparticle-based delivery systems have emerged as promising platforms for gut-specific targeting (220). These include lipid nanoparticles, polymer-based carriers, and biodegradable vesicles engineered to protect therapeutic cargo from enzymatic degradation in the gastrointestinal tract and promote uptake by mucosal immune cells (220).

Nano-drug delivery systems (NDDs) can be engineered to enhance mucosal adhesion (221), cellular uptake (222), and targeted delivery of antiretroviral agents or latency-reversing therapeutics (223) directly to infected cells in the gut. By bypassing efflux mechanisms and enabling sustained drug release (224), NDDs may achieve higher local drug concentrations and more effective suppression or elimination of HIV within tissue reservoirs. Incorporating targeting moieties, such as antibodies or ligands specific to infected cells (223) or the gut epithelium further enhances specificity and likely minimises off-target effects. Thus, NDDs represent a novel and rational approach to overcoming a key barrier in HIV cure strategies. Hydrogel-based (225) and mucoadhesive formulations (226, 227) offer additional avenues for localized delivery. These systems can be designed to release drugs in a sustained manner and enhance adhesion to the intestinal epithelium or Peyer’s patches, improving exposure to target cells while minimizing systemic absorption (228).

Antibody-drug conjugates (ADCs) are a class of precision therapeutics that employ monoclonal antibodies to selectively bind cell surface antigens, enabling targeted delivery of potent cytotoxic agents to tumour cells (229), including those in gastrointestinal cancers (230). Advancements in this field have led to the development of next-generation ADCs, such as bispecific ADCs, Probody-drug conjugates, immunostimulatory ADCs, degrader-antibody conjugates, and dual-payload ADCs (229). A variety of HIV-targeted (e.g., Env, Tat, Vif) and host-directed (e.g., CD25, CD4, CCR5, CXCR4, IL-2R) ADCs have been investigated, employing diverse payloads such as toxins, siRNAs, radionuclides, small molecule inhibitors, photosensitisers, and lipids (231). While clinical experience with HIV-specific ADCs remains limited, the field stands to benefit from advances in cancer immunotherapy (231). Targeting HIV-infected cells in the gut using ADCs represents a promising yet underexplored strategy, particularly given the tissue’s enrichment for latent reservoir cells and parallels with gastrointestinal cancer targeting.

Beyond anatomical targeting, delivery systems are being optimized to home to specific cellular reservoirs that represent major sources of persistent HIV in the gut (232). Ligand-conjugated nanoparticles are being engineered to exploit surface markers (e.g., integrins, chemokine receptors) expressed preferentially by target populations (233). These precision-targeting approaches aim to increase therapeutic efficacy while limiting off-target effects and could be applied in the context of HIV. As latency reversal and immunomodulation strategies progress toward clinical application, the integration of advanced delivery technologies will be critical to achieving therapeutic concentrations in gut tissues and enhancing the safety and specificity of HIV cure interventions.

## 4 Discussion

The gut constitutes one of the most formidable barriers to HIV eradication. As the largest immune organ in the body, it contains the vast majority of lymphoid tissue and CD4^+^ T cells (9), rendering it both a primary target for HIV infection and a long-lived viral reservoir. Despite the efficacy of ART in suppressing plasma viremia, HIV persists within GALT due to a convergence of structural, microbial, immunological, and pharmacological barriers. Cure strategies must therefore address not only systemic viral suppression but also the unique features of the gut reservoir, including its size, immune environment, and pharmacologic challenges.

Eliminating HIV reservoirs in the gut will likely require a multipronged therapeutic approach. Latency-reversing agents (LRAs) have shown partial activity in gut-derived cells, but their efficacy is limited by poor tissue penetration and lack of potency in reversing deep latency. Immune-based interventions, such as broadly neutralizing antibodies and immune checkpoint inhibitors, hold promise for enhancing reservoir clearance, but their ability to reach and act within GALT remains to be demonstrated. Drug delivery innovations, including nanoparticle formulations and tissue-targeted vectors, may help overcome the pharmacologic barriers posed by mucosal tissues, yet require rigorous evaluation in both preclinical and clinical settings.

In parallel, gene- and cell-editing approaches are emerging as transformative strategies for targeting gut HIV reservoirs. CRISPR-based interventions, such as EBT-101, and SIV-directed precursors have demonstrated broad biodistribution to lymphoid tissues, including the gut, with preclinical evidence of proviral excision. Although early clinical studies highlight the need for greater efficacy, these findings establish proof-of-concept that gene editing can indeed reach and act within GALT. Similarly, cell-based therapies, including CCR5-edited T cells and CAR-T platforms, offer the potential to repopulate the gut with resistant or effector cells capable of directly suppressing local HIV replication. The ability of engineered cells to traffic to and persist within mucosal tissues will be a critical determinant of their long-term success.

Strategies aimed at restoring gut barrier integrity and reducing inflammation, such as FMT, statins, anti-cytokine therapies, and gut-tropic agents, may act synergistically to suppress the drivers of HIV persistence. Therapeutic vaccines capable of eliciting robust mucosal CD8^+^ T cell responses are another key area under development, although translating these approaches into durable immune control remains a significant hurdle.

To overcome the anatomical and pharmacologic challenges of targeting the gut reservoir, innovative drug delivery systems, including ligand-targeted nanoparticles and mucoadhesive formulations, are under active investigation. These platforms may improve tissue penetration, increase drug stability, and allow for targeted delivery to infected cells within the mucosal environment.

Together, these efforts underscore the importance of integrating genetic engineering strategies with gut-specific delivery systems, immune modulation, and barrier-restoring interventions. The next phase of HIV cure research will require assessing not only the safety and durability of gene- and cell-editing therapies but also their functional impact on gut reservoirs. As such, trials should incorporate tissue-based endpoints, including gut biopsies and molecular reservoir profiling, to determine whether systemic interventions translate into meaningful reductions in mucosal reservoirs.

Looking ahead, several key knowledge gaps remain. The field would benefit from validated biomarkers of gut reservoir size and activity to assess therapeutic efficacy. Furthermore, most clinical trials do not include tissue-based endpoints, limiting our understanding of how interventions affect HIV persistence outside the peripheral blood. Longitudinal studies incorporating tissue pharmacokinetics, host immune responses, and microbiome dynamics are critical to informing rational therapeutic design. Ultimately, integration of multi-modal strategies targeting latency, inflammation, immune dysfunction, and mucosal damage will likely be necessary to achieve durable reductions in the gut HIV reservoir.

In conclusion, the gut represents a uniquely challenging and significant reservoir for latent HIV despite the clinical effectiveness of ART. Rational combination therapies, guided by mechanistic insights and empowered by advanced delivery platforms, offer a promising path forward in the endeavour to eliminate HIV reservoirs in the gut. However, success will depend on continued investment in tissue-based research and the development of clinical tools to measure and target HIV persistence at this critical site.
